# Editorial Note: *Lantana camara* plant extract catalyzed biosynthesis of graphene-metal nanocomposites with potential cytotoxic activities

**DOI:** 10.1371/journal.pone.0328186

**Published:** 2025-07-14

**Authors:** The PLOS One Editors

The *PLOS One* Editors issue this Editorial Note to inform readers that PLOS has concerns about the article’s authorship and about aspects of the article’s peer review. After reviewing this matter PLOS concluded that the article’s publication is supported based on expert input that was not affected by the concern(s).
